# Liquid Chromatography Coupled With High‐Resolution Mass Spectrometry Profiling of Metabolites in *Cassia alata* Flower Extracts and Their DPPH Free Radical Scavenging Activity

**DOI:** 10.1002/ansa.70114

**Published:** 2026-10-15

**Authors:** Adonai Yin‐Anaab Kunduzore, Shakira Ibrahim, Auphedeous Yinme Dang‐i, Michael Adu‐Frimpong, Salma Elshamy, Nikolai Kuhnert, James Ziemah

**Affiliations:** ^1^ School of Chemical and Biochemical Sciences University of Technology and Applied Sciences Navrongo Ghana; ^2^ Department of Pharmaceutical Biology German University in Cairo GUC New Cairo City Egypt; ^3^ School of Science Constructor University Bremen Bremen Germany; ^4^ Department of Chemistry and Pharmaceutical Sciences HSSCE Faculty Kingston University Kingston upon Thames UK

**Keywords:** antioxidant activity, *Cassia alata* flowers, flavonoids, LC‐HRMS, metabolite profiling

## Abstract

*Cassia alata* flowers remain phytochemically under‐characterized relative to the leaves. However, floral tissues can harbour distinct and, in some species, more abundant phenolic and flavonoid profiles, representing an unexploited source of antioxidant phytochemicals with potential functional‐food and nutraceutical relevance. Also, *Cassia alata* L. (Fabaceae) is a major source of numerous anthraquinones and flavonoids, yet the secondary metabolome of its floral tissues has not yet been thoroughly studied in comparison to its vegetative parts. This research therefore aimed to examine the phytoconstituents of *C. alata* flower extracts and evaluate their antioxidant potential. This represents, to our knowledge, the first liquid chromatography coupled with high‐resolution tandem mass spectrometry (LC‐HRMS/MS) and 2,2‐diphenyl‐1‐picrylhydrazyl (DPPH)‐based comparative profiling of *C. alata* flower extracts prepared with three solvents of differing polarity. Aqueous, 70% methanol, and 70% ethanol extracts of *C. alata* flowers were prepared and characterized using LC‐HRMS in negative ionization mode. The antioxidant potential was determined using the DPPH radical scavenging assay. LC‐HRMS enabled the tentative identification of 42 metabolites (in aqueous, methanolic, and ethanolic extracts), which were mainly flavonoids, phenolic acids, and glycosides. Some notable metabolites determined were Quercetin, Luteolin, Isomyricitin, Kaempferol‐dihexoside, Kaempferol‐*O*‐rutinoside, and Sophoricoside. The DPPH assay showed that all the extracts exhibited free radical scavenging activity in a concentration‐dependent manner. The ethanolic extract exhibited the strongest activity, with the lowest IC_50_ value of 12.10 ± 2.22 µg/mL, followed by the aqueous extract, while the methanolic extract showed the weakest activity among the plant extracts. The observed antioxidant potential of the ethanolic extract may be associated with the wide diversity of tentatively identified flavonoid‐related metabolites. These results suggest that *C. alata* flower extracts may serve as potential sources of antioxidant phytochemicals for food and health‐related applications.

## 1 Introduction

*Cassia alata* L. (Fabaceae) is a medicinal plant commonly referred to as Ringworm bush, Candle bush, *Senna alata*, among others. It has a widespread distribution throughout the tropical and subtropical parts of Africa, Asia, and South America [1]. Traditionally, it has been involved in the treatment of numerous diseases, among which are skin infections, inflammation, constipation, and oxidative stress‐related conditions. The primary use of *C. alata* L. involves its application to treat type 2 diabetes, heart disease, and many others [2, 3]. These ethnobotanical applications of *C. alata* have drawn more attention from the scientific community, especially in understanding its phytochemical composition and biological activity. In spite of this increased attention, majority of phytochemical and pharmacological studies have been on the leaves of the plant [4]. These studies have reported the presence of various bioactive constituents including phenols, flavonoids, and anthraquinones, which are known to contribute to the antioxidant and antimicrobial properties of the plant [5]. Nevertheless, other parts of the plant (such as the flowers) have not been well explored, even though they may also harbour unique and possibly useful metabolites.

Flowers are highly specialized plant tissues that tend to store unique secondary plant metabolites that engage in pigmentation, defence, and ecological interactions [6, 7]. It is increasingly evident that flowers can possess phenolic profiles different from those of leaves and seeds, which are usually characterized by high concentrations of flavonoids and phenolic acids [8]. These compounds have been recognized for having strong antioxidant activity and are increasingly significant in their applications in functional foods, natural preservatives, and dietary supplements [9, 10]. Flowers also play an important role in antioxidant activity due to their high levels of phenolic compounds, and in some cases, surpass the vegetative parts of the plant [11]. Irrespective of this information, the flowers of *C. alata* are underutilized, and little scientific information exists regarding their phytochemical composition and functional potential.

Among the most significant plant secondary metabolites are phenolic compounds because of their defence capacity and ability to scavenge reactive oxygen species (ROS) [2]. The antioxidant properties of phenolic compounds are important in preventing diseases, particularly chronic non‐communicable diseases such as cancer and type 2 diabetes [12, 13]. Plant products rich in polyphenols are very important in food science for substituting synthetic preservatives and enhancing the nutritional value of foods [9]. Earlier research has mainly described the presence of antioxidant activity in crude extracts of *C. alata*, which was most frequently determined using total phenol or total flavonoid content in the extract [14, 15]. Although these methods are useful in giving background information, they do not reveal the complexity of the phenolic composition or even the individual compounds that are responsible for this bioactivity [16]. Moreover, phytochemical research work conducted on *C. alata* has been mostly based on targeted or low‐resolution analysis techniques, which might overlook minor metabolites with significant biological relevance.

Liquid chromatography coupled with high‐resolution mass spectrometry (LC‐HRMS) is an effective method for untargeted and targeted metabolite profiling [17]. It is highly sensitive, accurate in mass measurements, and has greater structural elucidation capacity [18]. LC‐HRMS enables the identification of a large variety of metabolites, including flavonoids, phenolic acids, and their conjugates, even at low concentrations, with high mass accuracy and structural resolution [19]. These properties make LC‐HRMS particularly suitable for studying chemically rich plant tissues such as flowers.

Previous phytochemical studies of *C. alata* have mainly employed targeted chromatographic, spectrophotometric, or LC‐MS‐based approaches to characterize selected constituents or solvent fractions [15, 20]. However, comprehensive solvent‐comparative profiling of aqueous, methanolic, and ethanolic extracts of *C. alata* flowers using untargeted LC‐tandem HRMS (LC‐HRMS/MS) remains limited. Therefore, this study aimed to characterize the metabolite profiles of these three extracts using ultra‐HPLC (UHPLC)‐Orbitrap HRMS/MS and to evaluate their free‐radical scavenging activity using the DPPH assay. Metabolite annotations were cross‐validated using SIRIUS, GNPS, and PubChem with a mass‐accuracy criterion of <5 ppm. This approach provides a comprehensive assessment of the solvent‐dependent metabolite composition and antioxidant activity of *C. alata* flowers.

## 2 Materials and Methods

### 2.1 Chemicals and Materials

The chemicals utilized in the experimental pipeline were methanol (LC‐HRMS grade) and formic acid as HPLC grade for the mobile phase modifier and buffers (both purchased from Carl Roth, Karlsruhe, Germany), acetonitrile was used to measure the HPLC sequence (Acetonitrile for UHPLC gradient, J. T Baker, Gliwice, Poland), Milli‐Q water analyzed LC‐HRMS Reagent (J. T Baker, Gliwice, Poland), methanol and ethanol were used for extraction (both purchased from VWR International S. A. S, Rue Carrot Fontenay‐sous‐Bois, France), ascorbic acid and 2,2‐diphenyl‐1‐picrylhydrazyl (DPPH) (both purchased from Molychem, Badlapur, Maharashtra, India) were used for the antioxidant potential.

Fresh flowers of *C. alata* were collected from Navrongo (in the Upper East Region of Ghana) in the month of September during the rainy season. They are usually used as ornamental plants on the street. At the Department of Applied Biology in the University of Technology and Applied Sciences, this plant part was identified and authenticated by a botanist.

The flowers of *C. alata* were washed under running tap water, air‐dried, and powdered. The powder (100 g) was soaked in 1 L of aqueous methanol (70%) and ethanol (70%), each in a separate conical flask, and covered with aluminium foil. The mixtures were placed in an ultrasonicator at 40°C and sonicated for 1 h. The mixtures were further stirred overnight with the help of a magnetic stirrer for complete extraction. After that, the mixtures were centrifuged at 5000 rpm for 5 min to obtain the supernatant. The filtrates were concentrated under reduced pressure using a rotary evaporator at 55°C for 1 h.

### 2.2 Instrumentation and Experimental Conditions

LC‐HRMS analysis was performed according to the method described by [21], with slight modifications. LC‐HRMS analysis was carried out on a Thermo Vanquish system (Thermo Scientific, USA) equipped with a Poroshell 120 EC‐C18 column (2.7 µm, 2.1 × 100 mm, Agilent Technologies). The injection volume was 2 µL. A binary solvent system consisting of Milli‐Q water (Solvent A) and acetonitrile (Solvent B), both containing 0.01% formic acid (v/v), was employed. The flow rate was maintained at 0.4 mL/min, while the column temperature was set at 25°C.

The gradient elution program was as follows: starting at 8% B, linearly increased to 95% B over 23 min, held at 95% B until 33 min, then returned to 8% B by 40 min. The column was re‐equilibrated at 8% B for 10 min before the next injection.

The HPLC effluent was redirected to a Thermo Orbitrap IQ‐X mass spectrometer (Thermo Scientific) that had an electrospray ionization (ESI) source. Improved ionization parameters were established: vaporizer temperature, 350°C; sheath gas flow, 5.08 L/min; auxiliary gas flow, 12.11 L/min; and sweep gas flow, 2.72 L/min. Data collection was done in negative ion mode, with full scan MS [1] and MS/MS spectra. Negative ionization mode was selected because the major target classes, including flavonoids, phenolic acids, and their glycosides, readily undergo deprotonation during ESI (−). This mode can provide high sensitivity, low chemical background, and diagnostic fragmentation for these compounds, including retro‐Diels–Alder and glycosidic‐bond cleavages. The approach was also consistent with the previously reported method on which the analytical protocol was based [19]. However, the authors understand that exclusive use of ESI (−) may under‐represent metabolites that ionize preferentially in positive mode, including alkaloids and other amine‐containing compounds. Complementary ESI (+) analysis is therefore recommended in future studies to improve metabolome coverage, since this was out of the scope of this defined research.

The raw chromatographic data acquired by the mass spectrometer (MS) were converted to.mzML format using MSConvert in the ProteoWizard software suite. The raw MS data files were imported into MSConvert and converted to the open mzml file format for subsequent data processing and analysis. The mzML files were subsequently processed using MZmine to extract chromatographic features, including retention times, mass‐to‐charge ratios (m/z), and peak intensities. The processed data were further analyzed using SIRIUS and GNPS for compound annotation, with PubChem used to support compound identification. Subsequent data validation and chromatographic visualization were performed using FreeStyle and Quan Browser software (Thermo Fisher Scientific).

The DPPH scavenging activity was measured using a method by [22], with slight modification. Free radical scavenging was used to determine the antioxidant potential of the test extracts by checking the optical density of DPPH free radical. A DPPH solution with a concentration of 5.08 µM was prepared by dissolving 0.5 mg of DPPH in a volumetric flask containing methanol, prior to adjustment of the final volume to 250 mL. The resulting purple‐coloured DPPH free radical solution was stored for future use.

A mass of 10 mg of each crude extract was weighed into a 15 mL Falcon tube, and distilled water was added to reach the 10 mL mark. The samples were then vortexed to obtain a smooth, uniform mixture solution of 1000 µg/mL. Serial dilutions were prepared at concentrations of 5, 10, 20, 40, 60, 80, and 100 µg/mL from the stock solution (1000 µg/mL). This procedure was repeated for the standard ascorbic acid.

### 2.3 Optimized Final Method

A volume of 600 µL of the test extracts and the standard ascorbic acid of various concentrations was reacted with 1400 µL of 5.08 µM DPPH solution in a test tube. The mixture was then vortexed to ensure uniformity before being incubated in a dark chamber for 30 min. The samples were then transferred into the cuvettes, and the absorbance was measured at 517 nm using the UV‐Visible spectrophotometer (BIOBASE BK‐V1200, China). The samples were prepared in triplicate. The $IC_{50}$ (the concentration needed to scavenge or inhibit 50% of DPPH radical) was determined by plotting the linear regression of the % inhibition against the concentration. The percentage of the DPPH free radical scavenging activity was calculated using the following equation

$$\%\;\mathrm{inhibition}=\frac{\mathrm{abs}.\;\mathrm{of}\;\mathrm{control}-\mathrm{abs}.\;\mathrm{of}\;\mathrm{sample}}{\mathrm{abs}.\;\mathrm{of}\;\mathrm{control}}\;\times \;100$$

where %inhibition = antioxidant activity in percentage, abs. of control = absorbance of the control, and abs. of sample = absorbance of the sample.

### 2.4 Data Analysis

The experimental work was carried out in triplicate, and the data obtained were expressed as mean ± SD. Minitab 2022 was used to perform one‐way analysis of variance (ANOVA) and Tukey test analyses to compare the various plant extracts, while a significant difference was accepted at a *p*‐value of < 0.05. The graphical representation of the analysis was carried out using GraphPad Prism version 8.

## 3 Results and Discussion

### 3.1 LC‐HRMS Characterization of Compounds in Aqueous, Methanolic, and Ethanolic Extracts of the Sample

Database searches using SIRIUS, GNPS, and PubChem, following the approach described [23], enabled the identification of 42 compounds. Of these, 3 compounds were detected in both the aqueous and 70% methanol extracts. Twenty‐four compounds were detected only in the 70% methanol extract, while 4 compounds were unique to the 70% ethanol extract. The remaining 11 compounds were common to the 70% methanol and 70% ethanol extracts. Most compounds were tentatively annotated based on accurate mass measurements and MS/MS spectral matching against the reference databases. The chemical classification showed that the metabolome was dominated by phenolic compounds, especially flavonoids, phenols, and anthraquinones, which is in line with findings of [15], who worked on the leaf of *C. alata* and observed that the solvents used were the factor for this finding. Compounds with mass errors greater than 5 ppm were excluded from the tentative annotation. The complete 42‐compound dataset has been deposited in the Supporting Information (Table S1). The antioxidant‐relevant marker compounds, their retention times, molecular formulae, mass errors, and fragment‐ion data are provided in Table S1. The chemical structures and chromatograms are presented in Figures 1 and S1, respectively. Details of the compounds are listed in Table S1.

Isomyricitrin (*m/z* 479.0820; $C_{21}H_{20}O_{13}$), which was identified in the aqueous and 70% methanol extracts, eluted at 15.53 min, with characteristic fragment ions at 299.0200 and 279.1241. Similarly, Luteolin profiled in the 70% methanol extract occurred at 38.68 min with an *m/z* peak of 285.0398 ($C_{15}H_{10}O_{6}$) and fragment ions at 267.0287 and 112.6352. Additionally, the 70% methanol extract displayed an ESI‐MS signal at 38.76 min, which was tentatively identified as Quercetin (*m/z* 301.0343; $C_{15}H_{10}O_{7}$) with fragment ions of 151.0032 and 273.0396. Moreover, Scophoricoside (*m/z* 431.0973; $C_{21}H_{20}O_{10}$) was found to be present in both 70% methanol and 70% ethanol extracts, eluted at 14.89 min, and showed characteristic fragment ions of 89.0231 and 71.0133. The tentatively identified compounds have not been profiled in the *C. alata* flower extract. However, these compounds have been reported to have strong antioxidant capabilities in some plant extracts. In particular, Isomyricitrin has been isolated from *Syzygium samarangense* and evaluated to have strong antioxidant potential owing to its ability to protect keratinocytes against oxidative stress [24]. It has also been previously reported that Quercetin and Luteolin were identified in the leaf of *C. alata* [25]. Quercetin and Luteolin in particular have also been isolated from other plant extracts and have been shown to exhibit strong antioxidant potential [28, 29]. The flavonol‐ and flavone‐dominated profile of the flower contrasts with the anthraquinone‐rich profiles commonly reported for *C. alata* leaves. This suggests that the flowers represent a phytochemically distinct and complementary source of bioactive metabolites [24, 25, 26, 27]. Sophorcoside, a bioactive compound found in a medicinal herb (*Sophora japonica*), was able to prevent oxidative stress and nuclear factor‐Kappa B activation in mice with ConA‐induced hepatitis [30]. Herein, all these notable compounds tentatively identified in *C. alata* extract could be attributed to the extract's antioxidant potential observed in the in vitro DPPH assay. The presence of these compounds may contribute to hydrogen atom‐donating and electron‐donating activities, chelating, and inhibitory activity of oxidative chain reactions in this extract [31]. This makes *C. alata* flower extracts a valuable source of bioactive compounds, particularly phenolic compounds, and relevant for health benefits.

Also, the structure‐activity relationships may partly explain the differences in radical‐scavenging activity among the extracts. The 3′,4′‐dihydroxy (catechol) B‐ring and the C2 = C3 bond conjugated with the 4‐oxo group are important determinants of flavonoid antioxidant activity. These structural features facilitate hydrogen or electron donation and stabilize the resulting flavonoid radicals [32, 33]. Quercetin and luteolin possess both features, while myricetin has an additional 5′‐hydroxyl group on the B‐ring. The influence of this additional hydroxyl group can vary with the antioxidant assay and experimental conditions [34]. In contrast, O‐glycosylation can reduce radical‐scavenging activity relative to the corresponding aglycones by altering electron delocalization and limiting the accessibility of reactive hydroxyl groups [21, 32]. This effect may partly contribute to the higher IC_50_ values observed for extracts containing greater proportions of glycosylated flavonoids. However, the antioxidant activity of each extract likely reflects the combined effects and relative abundances of multiple constituents.

The MS/MS assignments were based on established flavonoid fragmentation patterns. Neutral losses of 162, 146, 132, and 176 Da were used as diagnostic evidence for hexose, deoxyhexose, pentose, and glucuronic acid conjugates, respectively. Retro‐Diels–Alder fragmentation of the flavonoid skeleton provided additional structural information for flavone and flavonol aglycones [35]. Structures of some detailed tentatively identified compounds in the extracts are shown in Figure 1. The chromatograms for the extracts are also illustrated in the supporting information sheet in Figure S1.

### 3.2 Antioxidant Activity

The 70% methanol extract showed the most complex profile, containing 38 of the 42 annotated metabolites, including 24 unique compounds. The aqueous extract was dominated by highly polar metabolites, whereas the 70% ethanol extract contained fewer compounds (15 of 42) but was relatively enriched in less‐glycosylated flavonoids and aglycones. The presence of these compounds may contribute to the stronger radical‐scavenging activity reported below, suggesting that antioxidant activity depends on metabolite composition rather than overall metabolite richness. Several annotated metabolites eluted within the first 1–2 min of the chromatographic gradient. These early‐eluting features were mainly highly polar compounds, including free sugars, sugar‐conjugated metabolites, and organic acids. Such compounds are poorly retained and difficult to resolve using reversed‐phase C18 chromatography and may be susceptible to ion suppression from co‐eluting matrix components [36]. To minimize misassignment, metabolite annotation was based on accurate mass (<5 ppm) and MS/MS fragmentation rather than retention time alone. Complementary hydrophilic interaction liquid chromatography is recommended for improved separation and characterization of this highly polar fraction in future studies [37]. The free radical inhibitory effect of the extracts (aqueous, 70% methanol, and 70% ethanol) and ascorbic acid was measured between 5 and 100 µg/mL. It was established that all the extracts exhibited enhanced activity with increasing concentration. The ability of ascorbic acid to inhibit more effectively at all concentrations makes it a potent reference inhibitor. The ethanolic extract showed a considerably better inhibitory activity than the aqueous and methanolic extracts at all tested concentrations.

These findings were further supported by the $IC_{50}$ values Figure 2. The lowest $IC_{50}$ (5.94 ± 0.64 µg/mL) was exhibited by ascorbic acid, which is an indication of its greater inhibitory effect as a control. Among the extracts, the 70% ethanol extract had the lowest $IC_{50}$ value (12.10 ± 2.22 µg/mL), followed by the aqueous extract, which had an $IC_{50}$ value of 17.57 ± 2.51 µg/mL, and the 70% methanol extract, which had an $IC_{50}$ value of 71.37 ± 3.95 µg/mL. An extract with $IC_{50}$ values ranging between 10 and 50 µg/mL exhibits strong antioxidant potential, and between 50 and 100 µg/mL exhibits intermediate antioxidant potential [38].

**FIGURE 1:**
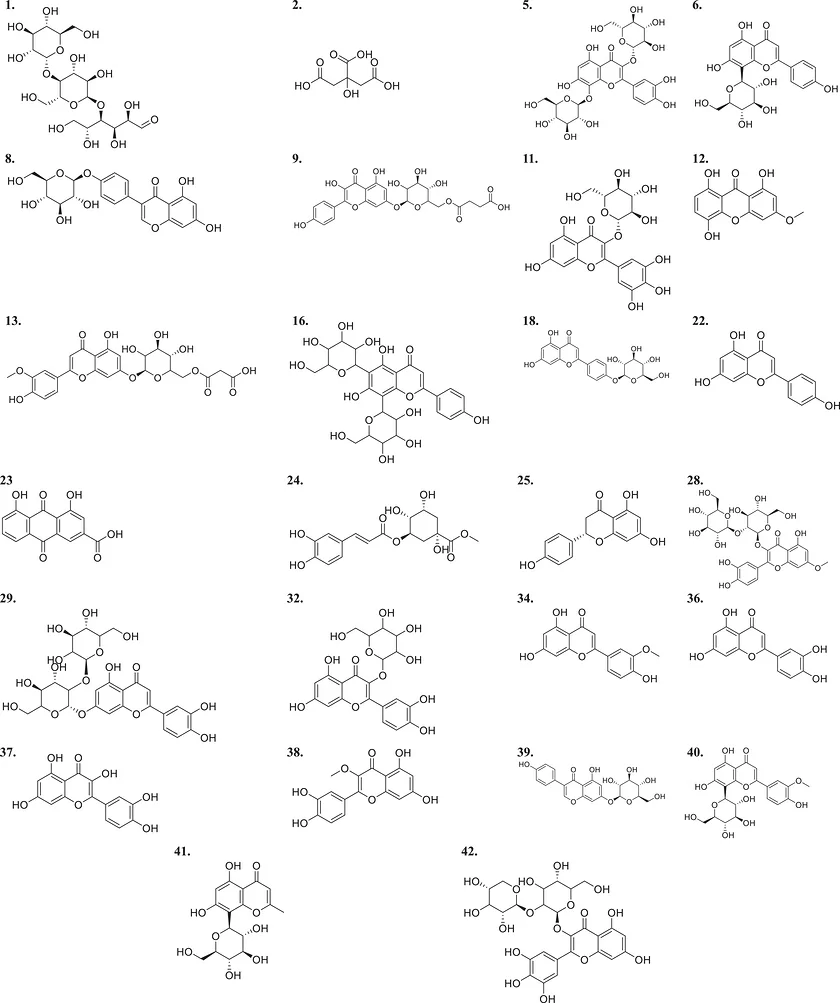
Chemical structures of selected phytochemicals tentatively annotated in *C. alata* flower extracts. Details of the phytochemicals including fragmentations, theorical and experimental M/Z are shown in Table S1.

**FIGURE 2:**
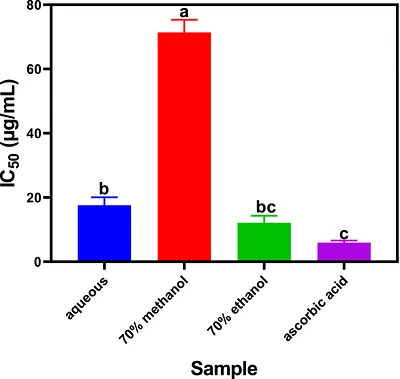
DPPH Antioxidant Values of aqueous, 70% methanol, 70% ethanol extracts, and ascorbic acid against 2,2‐diphenyl‐1‐picrylhydrazyl (DPPH) radicals. The results are indicated as the mean and standard deviation (SD); the means that do not have a common letter indicate significant differences with *p* < 0.05.

Based on the principle of IC_50_, 70% ethanol and the aqueous extract demonstrated strong antioxidant potential, while the 70% methanol extract exhibited an intermediate antioxidant potential. This study also compares with and confirms a previous study on the leaf extract of *C. alata* with $IC_{50}$ value of 54 ± 2.20 µg/mL [39]. In another study, the $IC_{50}$ values of the leaves, roots, and twigs of *C. alata* extracted with acetone were 41.80, 29.51, and 26.23 µg/mL, respectively [40]. The presence of high concentrations of flavonoids and phenolic compounds in the 70% ethanol and aqueous extracts of *C. alata* flower might therefore explain their significant scavenging ability [41]. The identified polyphenols can scavenge free radicals and modulate ROS, which are implicated in the development of several chronic diseases [39]. This supports a potential association between the LC‐HRMS‐annotated metabolites and the antioxidant activity of the extracts. One‐way ANOVA followed by Tukey's post‐hoc test showed significant differences among the IC_50_ values of the three extracts (*p* < 0.05; Figure 2). A limitation of this study is that antioxidant activity was evaluated using only the DPPH assay. DPPH primarily assesses radical‐scavenging capacity through electron‐ and hydrogen‐atom‐transfer mechanisms [42]. Complementary methods, such as FRAP for reducing capacity and ABTS for radical‐scavenging activity, would provide further assessment confirmation in the future [42]. Future studies should combine multiple antioxidant assays with total phenolic and total flavonoid contents and evaluate their correlations with the metabolite profiles. Our results also highlight the significance of the choice of solvent in the maximization of bioactivity and justify the possibility that ethanol‐extracted phytochemicals can be used as natural inhibitors against oxidative stress (Figure 2).

## 4 Conclusion

This study provides the first LC‐Orbitrap‐HRMS/MS‐based, multi‐database‐validated metabolite profile of *C. alata* flowers across three extraction solvents, together with a bioactivity‐guided DPPH assessment. LC‐HRMS profiling showed tentative identification of multiple phenolic acids, flavonoids, and other related secondary metabolites totalling 42. The extracts also showed potential antioxidant effects, indicating that *C. alata* flower extracts, which contain a wide range of chemical compounds, are valuable sources of antioxidants. The *C. alata* flower extracts had a concentration‐dependent inhibitory effect, with the most potent and lowest $IC_{50}$ observed in the ethanolic extract compared to the control. The heightened scavenging activity of the ethanolic extract can be attributed to its abundance and diversity of bioactive constituents identified in this study, such as Isomyricitrin, Quercetin, Luteolin, Kaempferol‐O‐rutinoside, and Sophoricoside. In general, the *C. alata* flower offers a promising source of antioxidant‐related phytochemicals. This study provides a phytochemical foundation for the potential use of these compounds as functional foods and health‐related applications.

## Author Contributions

**Adonai Yin‐Anaab Kunduzore**: conceptualization, methodology, investigation, validation, writing – original manuscript, draft, and review; **Shakira Ibrahim**: conceptualization, methodology, investigation, and review; **Auphedeous Yinme Dang‐i**: conceptualization, validation, supervision, and review; **Michael Adu‐Frimpong**: supervision and review; **Salma Elshamy**: writing – original manuscript, supervision, and review; **Nikolai Kuhnert**: supervision and review; **James Ziemah**: conceptualization, validation, writing – original manuscript, draft, supervision, and review.

## Funding

Funding was provided by the University of Technology and Applied Sciences and Kingston University, London.

## Conflicts of Interest

The authors declare no conflicts of interest.

## Supporting information




**Supporting File**: ansa70114‐sup‐0001‐SuppMat.docx.

## Data Availability

The data supporting the findings of this study are available within the article and its supplementary information files. Raw LC‐HRMS/MS data are available with the corresponding author upon request. Additional LC‐HRMS raw data may be made available by the corresponding authors upon request.
