# Supplementary material for: Liquid Chromatography Coupled With High‐Resolution Mass Spectrometry Profiling of Metabolites in Cassia alata Flower Extracts and Their DPPH Free Radical Scavenging Activity
Source: Anal Sci Adv. 2026 Oct 15;7(2):e70114. doi: 10.1002/ansa.70114 (PMC13671258; doi:10.1002/ansa.70114)
Supplement: Supplementary file 1 [file ANSA-7-e70114-s001.docx]

**LC-HRMS Profiling of Metabolites in *Cassia alata* Flower Extracts and Their DPPH Free Radical Scavenging Activity**

Adonai Yin-Anaab Kunduzore^2^^, ℸ^, Shakira Ibrahim^2, ℸ^, Auphedeous Yinme Dang-i^2*^, Michael Adu-Frimpong^2^, Salma Elshamy^4^, Nikolai Kuhnert^3^, James Ziemah^1, 2, 3 *^

1. Department of Chemistry and Pharmaceutical Sciences, HSSCE Faculty, Kingston University, Kingston-Upon-Thames, Surrey, KT1 2EE, UK
2. School of Chemical and Biochemical Sciences, University of Technology and Applied Sciences, Navrongo, Ghana
3. School of Science, Constructor University Bremen, 28759 Bremen, Germany
4. Department of Pharmaceutical Biology, German University in Cairo GUC, 11835 New Cairo City, Cairo, Egypt

*Corresponding authors: [J.Ziemah@kingston.ac.uk](mailto:J.Ziemah@kingston.ac.uk)

**^ℸ^** These authors contributed equally to this work.

**Figure S1.** Liquid chromatography coupled with high-resolution mass spectrometry chromatogram of extracts in negative ion mode: (a) aqueous, (b) 70% methanol, and (c) 70% ethanol with details in **Table S1**. All plots represent the base peak intensity versus retention time.

**Table S1.** Complete list of 42 tentatively annotated compounds from aqueous, 70% methanol, and 70% ethanol extracts of Cassia alata flowers by LC-HRMS with fragments in negative mode. RT = retention time, Err = error, ppm = parts per million, m/z = mass to charge ratio. H = compounds found in aqueous extract, M = compounds found only in 70% methanol extract, E = compounds found only in 70% ethanol extract, HM = compound found in aqueous and 70% methanol extracts, ME = compound found in 70% methanol and 70% ethanol extracts.

| **#** | **RT (min)** | **Molecular formula** | **Err (ppm)** | **Theoretical m/z** | **Experimental m/z** | **Compound Name** | **Fragment ion 1** | **Fragment ion 2** | **Fragment ion 3** |
| --- | --- | --- | --- | --- | --- | --- | --- | --- | --- |
| 1 | 1.56 | C₁₈H₃₂O₁₆ | 5.18 | 503.1607 | 503.1627 | Maltotriose^ME^ | 71.0135 |  |  |
| 2 | 1.92 | C_6_H_8_O_7_ | 1.77 | 191.0186 | 191.019 | Citric acid^M^ | 173.0090 | 111.0080 |  |
| 3 | 11.66 | C_27_H_30_O_15_ | -1.87 | 593.1501 | 593.149 | Apigenin-O-deoxyhexosyl-hexoside^M^ | 269.0443 |  |  |
| 4 | 14.10 | C_27_H_30_O_15_ | -1.15 | 593.1501 | 593.1494 | Apigenin-O-deoxyhexosyl-hexoside^M^ | 269.0443 |  |  |
| 5 | 12.92 | C₂₇H₃₀O₁₈ | -2.81 | 641.1348 | 641.1371 | Gossypetin-3, 8-O-diglucosideME | 207.0449 |  |  |
| 6 | 14.20 | C_21_H_20_O_10_ | -0.81 | 431.0973 | 431.0969 | Vitexin^M^ | 269.0451 | 174.4906 |  |
| 7 | 11.54 | C_21_H_20_O_10_ | -0.59 | 609.1501 | 609.1447 | Vitexin^M^ | 446.0818 | 284.0325 |  |
| 8 | 14.89 | C₂₁H₂₀O₁₀ | 0.62 | 431.0973 | 431.097 | Sophoricoside^ME^ | 89.0239 | 71.0133 | 113.0239 |
| 9 | 15.08 | C₂₅H₂₄O₁₄ | 3.43 | 547.1082 | 547.1101 | Kaempferol 7-(6''-p-succinylglucoside)^M^ | 431.0989 | 193.9268 | 116.9287 |
| 10 | 15.50 | C₁₄H₈O₅ | -2.05 | 255.0288 | 255.0294 | 1,4,5-trihydroxy-9,10-dihydroanthracene-9,10-dione^HM^ | 167.0499 |  |  |
| 11 | 15.53 | C₂₁H₂₀O₁₃ | 3.47 | 479.082 | 479.0837 | Isomyricitrin^HM^ | 299.0200 | 279.1241 | 116.9287 |
| 12 | 15.60 | C_14_H_10_O_6_ | -0.88 | 273.0394 | 273.0391 | BellidifolinM | 255.0293 |  |  |
| 13 | 16.13 | C₂₅H₂₄O₁₄ | 3.87 | 547.1082 | 547.1104 | Luteolin 3'-methyl ether 7-malonylglucoside^ME^ | 431.0992 | 279.0655 | 225.0557 |
| 14 | 19.01 | C₂₅H₂₄O₁₄ | 3.87 | 547.1082 | 547.1104 | Luteolin 3'-methyl ether 7-malonylglucoside^ME^ | 431.0992 | 279.0655 | 225.0557 |
| 15 | 19.83 | C₂₅H₂₄O₁₄ | 3.43 | 547.1082 | 547.1101 | Luteolin 3'-methyl ether 7-malonylglucoside^ME^ | 431.0988 | 329.4080 |  |
| 16 | 17.67 | C₂₇H₃₀O₁₅ | -0.84 | 593.1501 | 593.1496 | Violantin^ME^ | 383.0766 | 225.0548 |  |
| 17 | 18.56 | C_21_H_21_O_9_ | 1.84 | 417.11801 | 417.1188 | Liquiritigenin O-hexoside^M^ | 327.0777 | 255.0640 |  |
| 18 | 19.89 | C_21_H_20_O_10_ | -2.16 | 431.0973 | 431.0963 | Apigenin 4'-O-beta-D-glucopyranoside^ME^ | 341.0654 | 269.0445 |  |
| 19 | 19.30 | C_21_H_20_O_10_ | 0.25 | 431.09727 | 431.0974 | Apigenin 4'-O-beta-D-glucopyranoside^ME^ | 269.0444 |  |  |
| 20 | 16.04 | C_21_H_20_O_10_ | -0.67 | 431.0973 | 431.097 | Apigenin 4'-O-beta-D-glucopyranoside^ME^ | 269.0443 |  |  |
| 21 | 16.04 | C_21_H_20_O_10_ | -0.10 | 431.0973 | 431.0972 | Apigenin 4'-O-beta-D-glucopyranoside^ME^ | 269.0443 |  |  |
| 22 | 19.96 | C_15_H_10_O_5_ | 3.91 | 269.0445 | 269.0455 | Apigenin^M^ | 151.6487 | 107.7080 |  |
| 23 | 20.47 | C_15_H_8_O_6_ | 2.15 | 283.2370 | 283.0243 | Rhein^M^ | 239.0343 | 144.1180 |  |
| 24 | 21.60 | C_16_H_20_O_7_ | -0.13 | 323.1125 | 323.1125 | Methyl chlorogenate^M^ | 191.0336 | 165.0529 |  |
| 25 | 22.59 | C_15_H_12_O_5_ | 1.76 | 271.0601 | 271.0606 | Naringenin^M^ | 153.9087 |  |  |
| 26 | 22.67 | C_27_H_30_O_16_ | -0.29 | 609.145 | 609.1448 | Kaempferol dihexoside^M^ | 284.0315 |  |  |
| 27 | 11.54 | C_27_H_30_O_16_ | -0.59 | 609.1501 | 609.1447 | Kaempferol dihexoside^M^ | 446.0818 | 284.0325 |  |
| 28 | 23.24 | C₂₈H₃₂O₁₇ | -1.05 | 639.1556 | 639.158 | Rhamnetin 3-sophoroside^M^ | 476.0963 | 313.0368 |  |
| 29 | 24.47 | C₂₇H₃₀O₁₆ | 2.82 | 609.1450 | 609.1467 | Luteolin 7-allosyl-(1->2)-glucoside^M^ | 327.0500 | 311.0568 |  |
| 30 | 24.91 | C_27_H_29_O_15_ | -0.22 | 593.1500 | 593.1500 | Kaempferol-O-rutinoside^M^ | 284.0307 |  |  |
| 31 | 25.50 | C_28_H_31_O_16_ | 0.85 | 623.1607 | 623.1612 | Isorhamnetin-O-rutinoside^M^ | 314.0433 |  |  |
| 32 | 28.76 | C_21_H_20_O_11_ | -3.89 | 447.0922 | 447.0939 | Quercetin-O-hexoside^M^ | 300.9195 |  |  |
| 33 | 28.80 | C_21_H_20_O_11_ | -0.06 | 447.0922 | 477.0922 | Kaempferol-O-hexoside^M^ | 284.0316 |  |  |
| 34 | 35.99 | C_16_H1_2_O_6_ | -1.71 | 299.055 | 299.0545 | Chrysoeriol^M^ | 255.0281 | 227.0327 |  |
| 35 | 36.20 | C_31_H_28_O_11_ | 0.42 | 575.1548 | 557.155 | Apigenin-O-rutinoside^M^ | 269.0440 | 255.0550 |  |
| 36 | 38.68 | C₁₅H₁₀O₆ | 1.51 | 285.0394 | 285.0398 | Luteolin^M^ | 267.0287 | 112.6352 |  |
| 37 | 38.76 | C₁₅H₁₀O₇ | 4.94 | 301.0343 | 301.0358 | Quercetin^M^ | 151.0032 | 273.0396 |  |
| 38 | 39.19 | C₁₆H₁₂O₇ | 3.29 | 315.0499 | 315.051 | 3-O-Methylquercetin^M^ | 174.9562 | 85.9273 |  |
| 39 | 16.04 | C₂₁H₂₀O₁₀ | 0.236 | 431.09837 | 431.09847 | Genistin^E^ | 269.0446 | 225.0555 | 311.0568 |
| 40 | 26.48 | C₂₂H₂₂O₁₁ | -3.806 | 461.10894 | 461.10718 | Scoparin^E^ | 341.0653 | 353.0137 |  |
| 41 | 12.53 | C₁₆H₁₈O₉ | -2.862 | 353.08781 | 353.08680 | Isobiflorin^E^ | 233.0446 | 263.0557 |  |
| 42 | 13.96 | C₂₆H₂₈O₁₇ | -2.762 | 611.12537 | 611.12369 | Myricetin 3-sambubioside^E^ | 255.0287 | 299.0194 |  |
